# Comparison of Analgesic Effect between Gabapentin and Diclofenac on Post-Operative Pain in Patients Undergoing Tonsillectomy

**DOI:** 10.5812/atr.10117

**Published:** 2013-08-01

**Authors:** Visnja Nesek Adam

**Affiliations:** 1 University Department of Anesthesiology, Resuscitation and Intensive Care, University of Osijek, Osijek, Croatia

**Keywords:** Pain, Gabapentin, Diclofenac, Tonsillectomy

Dear Editor,

Concerning the article “Comparison of Analgesic Effect between Gabapentin and Diclofenac on Post-Operative Pain in Patients Undergoing Tonsillectomy” published in one of the previous issues of Archives of Trauma Research (1), I would like to sincerely congratulate the authors on the results obtained in their study.

Tonsillectomy is an operation that is unpleasant and deeply affects the patient, especially children, for several days after surgery. Although, a variety of different types of drugs have been used for pain relief following tonsillectomy, it is still not effectively and fully controlled. Gabapentin is mostly used to treat chronic pain, but an increasing number of randomized trials demonstrated its efficacy in treating postoperative pain and reducing opioid consumption (2). However, evidence for the effectiveness of gabapentin in patients undergoing tonsillectomy is limited to a small number of studies (3, 4). Although this study showed similar efficacy of diclofenac and gabapentin in post-tonsillectomy pain, this research is very important, because it supports the use of gabapentin as a relatively new therapeutic option for postoperative pain control and demonstrates promising results in reducing pain after tonsillectomy.

This was a carefully performed and well written study. The discussion is very good. However, there are several issues that need to be addressed. First, the results were calculated using repeated student’s t-tests for the parametric variables. The question is whether this is the most appropriate test to compare the three groups and whether it would change the conclusions if the results are analyzed differently? In this regard, it would be advised to use ANOVA or more suitable, to use repeated measures ANOVA. After that, the primary and secondary outcomes of interest and the time point used are not clear.

Lastly, although the authors have focused on the analgesic effect of gabapentin and diclofenac, it would be important and very engaging to discuss the side effects. The interesting aspect of these results is the proportion of patients experiencing vomiting, which is not remarkable in the present study despite the "emetic nature" of tonsillectomy surgery. Side effects of gabapentin included vomiting, dizziness and headache, but these side effects are usually seen in long term infusion of gabapentin or high doses of gabapentin administration. However, a recently published clinical study demonstrated that gabapentin is effective for reducing the nausea and vomiting induced by chemotherapy (5) and surgeries like open and laparoscopic cholecystectomy (6, 7). In this regard, the low proportion of patients experiencing vomiting in the gabapentin group could be explained by the claimed antiemetic effect of gabapentin but low proportion of patients in the placebo and diclofenac groups remains to be determined. It would be interesting to conduct future studies with larger patient series to better define the antiemetic efficacy of gabapentin and thus the possible advantage of gabapentin compared to diclofenac.

And finally, once again I would like to congratulate the authors for such an interesting and comprehensive study.
